# Examining group differences in between-participant variability in non-native speech sound learning

**DOI:** 10.3758/s13414-021-02311-3

**Published:** 2021-06-04

**Authors:** Pamela Fuhrmeister

**Affiliations:** Department of Linguistics, University of Potsdam, Karl-Liebknecht-Straße 24-25, 14476 Potsdam, Germany

**Keywords:** Non-native speech sound learning, Individual differences, Heterogeneity of variance

## Abstract

Many studies on non-native speech sound learning report a large amount of between-participant variability. This variability allows us to ask interesting questions about non-native speech sound learning, such as whether certain training paradigms give rise to more or less between-participant variability. This study presents a reanalysis of Fuhrmeister and Myers (*Attention, Perception, and Psychophysics, 82*(4), 2049-2065, 2020) and tests whether different types of phonetic training lead to group differences in between-participant variability. The original study trained participants on a non-native speech sound contrast in two different phonological (vowel) contexts and tested for differences in means between a group that received blocked training (one vowel context at a time) and interleaved training (vowel contexts were randomized). No statistically significant differences in means were found between the two groups in the original study on a discrimination test (a same-different judgment). However, the current reanalysis tested group differences in between-participant variability and found greater variability in the blocked training group immediately after training because this group had a larger proportion of participants with higher-than-average scores. After a period of offline consolidation, this group difference in variability decreased substantially. This suggests that the type and difficulty of phonetic training (blocked vs. interleaved) may initially give rise to differences in between-participant variability, but offline consolidation may attenuate that variability and have an equalizing effect across participants. This reanalysis supports the view that examining between-participant variability in addition to means when analyzing data can give us a more complete picture of the effects being tested.

Many learning studies test for differences in group means as a result of some intervention: A study is typically designed so that a group of learners receives some type of treatment and they are compared to a control group or a group receiving another type of treatment. If the group receiving one type of intervention has a higher mean than a control group or the group receiving another type of intervention, the intervention is considered successful. Often times, researchers test for heterogeneity of variance to ensure their data meet assumptions of *t* tests or ANOVAs, and they typically view heterogeneity of variance as a nuisance that needs to be dealt with in order to test for group differences in means. While differences in means are certainly appropriate and meaningful for many questions, there is a wealth of information to be gained from testing for group or individual differences in variance or variability more generally. For instance, Bryk and Raudenbush (1988) argue that heterogeneity of variance between groups after a treatment indicates the presence of an interaction with some unmeasured variable. They additionally discuss two different scenarios: equalizing and disequalizing treatments. Disequalizing treatment effects occur when high-ability learners benefit disproportionally from a treatment compared to low-ability learners. This results in increased variance in the group that received the disequalizing treatment. In contrast, equalizing effects describe treatment effects that confer a larger benefit to low-ability learners, therefore decreasing the between-participant variability observed. These are interesting and important effects to consider, but we can only consider them if we explicitly analyze between-participant variability.

Non-native speech sound learning is a type of learning that seems to be particularly susceptible to individual differences, especially for adult learners (e.g., Bradlow, Pisoni, Akahane-Yamada, & Tohkura, 1997; Bradlow, Akahane-Yamada, Pisoni, & Tohkura, 1999; Golestani & Zatorre, 2004; Golestani & Zatorre, 2009; Myers & Swan, 2012; Perrachione, Lee, Ha, & Wong, 2011; Lim & Holt, 2011; Luthra et al., 2019). In fact, almost all published studies on non-native speech sound learning report a large amount of between-participant variability, or this can be seen from the wide range in scores or standard errors in the data. This variability between learners offers unique and interesting opportunities to discover more about the process of learning new speech sounds. For instance, with a correlational approach, we can find out which skills or other variables are related to successful non-native speech sound learning. Tasks that produce a large amount of between-participant variability are particularly well suited to correlational research because they are better able to rank participants in their performance compared to tasks that produce limited variability between participants (Hedge, Powell, & Sumner, 2018). Another question we can ask is whether this between-participant variability is systematic in some way. For example, it is possible that learners generally improve at the same rate from training, and individual differences result simply from pre-existing differences in aptitude. Another possibility is that individuals benefit differently from different types of training. In other words, some individuals might experience a disproportionate benefit from a certain type of training, while others see little to no benefit of that type of training.

Some studies in the non-native speech sound learning literature suggest that participants may benefit differently or unequally from certain types of training by showing that learner aptitude interacts with training type. For example, a few studies that have trained listeners to distinguish non-native lexical tones have used high-variability phonetic training (training in which learners are exposed to speech sounds produced by several different talkers, or the sounds are presented in multiple phonological contexts). Two of these studies found that success with high-variability training (compared with low-variability, i.e., hearing one talker in training) depends on a learner’s aptitude (Sadakata & McQueen, 2014; Perrachione, Lee, Ha, & Wong, 2011). Perrachione, Lee, Ha, and Wong (2011) found that this was specifically due to the *presentation* of the variability in the stimulus set: Low-aptitude learners could benefit from high-variability training if different talkers were presented in a blocked manner in training (i.e., one talker at a time), as opposed to an interleaved manner (trials with different talkers are presented randomly). A similar non-native tone learning study, however, did not find evidence that learner aptitude predicted success with high-variability training for either blocked or interleaved presentation of the variability; however, a Bayes factor analysis indicated that their data were inconclusive for this particular effect and did not substantially favor the null over the alternative hypothesis (Dong, Clayards, Brown, & Wonnacott, 2019).

Fuhrmeister and Myers (2020) similarly manipulated the presentation of variability (blocked vs. interleaved presentation of phonological contexts) in their stimulus set to train listeners to learn a non-native segmental contrast, though this study differed from the previous ones discussed here in that they only used a limited amount of total variability in the stimulus set (i.e., this cannot be considered a high-variability training study). Nonetheless, they found that higher pre-training aptitude predicted success with interleaved phonetic training on an identification task (though not a discrimination task). These findings provide some evidence that presenting talker or phonological variability in an interleaved manner may be a type of disequalizing treatment in non-native speech sound learning, in that high-aptitude learners benefit disproportionately. However, blocked training may be an equalizing treatment, also allowing low-aptitude learners to learn. A way to test this directly would be to test for differences in between-participant variability between groups receiving blocked and interleaved training. Though previous studies have tested for group differences in means, it is possible that we can uncover even more information by testing for group differences in variability. For example, two groups may not show statistically significant differences in means if the variance in one or more groups is too large. However, the variance of one group may be larger than the other, which could suggest an interaction with another variable that was not measured.

## Current study

The current study presents a reanalysis of the data from the discrimination task^1^ from Fuhrmeister and Myers (2020) to test for group differences in between-participant variability in blocked vs. interleaved training groups. The original study trained native English speakers to learn the voiced dental and retroflex stop consonants in Hindi and presented the sounds in two different phonological contexts. In each context, the sounds to be learned were presented in word-initial position but followed by one of two vowel contexts: /i/ or /u/. One group of participants received training on the sounds in a blocked manner (a block of the /u/ vowel context, followed by a block of the /i/ context), and one group received interleaved training (vowel contexts were intermixed during training). The original study tested for group differences in *means* and found no significant differences between the two groups on the discrimination task.

If interleaved training is a disequalizing treatment, we would expect to see greater between-participant variability in the interleaved group after training than the blocked group. In the original study, we tested participants’ discrimination of the sounds at three time points: prior to training (pretest), immediately after training (to assess immediate learning), and again the next morning after a period of offline consolidation (to assess retention after a delay). The current reanalysis tests for training group (blocked vs. interleaved) differences in between-participant variability at each of those time points, and we predict that interleaved training will result in more between-participant variability after training than the blocked group.

It is also possible that offline consolidation of the learned speech sounds will act as an equalizing factor. For example, several studies have found that learners improve on perceptual speech sound learning tasks after sleep (e.g., Earle, Landi, & Myers, 2017; Fenn, Nusbaum, & Margoliash, 2003), so it is possible that some learners will perform better on non-native speech sound learning tasks after the learned phonetic information has been consolidated. This prediction is also supported by other theories of learning. For example, a theory called the contextual interference effect (a theory of learning originally applied to motor learning) predicts that interleaved learning results in poorer performance during learning (due to increased difficulty) but superior performance when retention or generalization is tested (e.g., Battig 1972; Magill & Hall 1990; Shea & Morgan 1979). This theory could be extended to make predictions for the current reanalysis: Interleaved training may result in greater between-participant variability immediately after training, especially if certain learners, such as high-aptitude learners, show a greater benefit (e.g., Perrachione et al., 2011). According to the contextual interference effect, however, interleaved training should confer delayed benefits to learners. In that case, we might expect some learners to “catch up” after a period of offline consolidation, therefore decreasing the between-participant variability.

## Methods

Complete details of the methods can be found in the original study (Fuhrmeister & Myers, 2020), but a summary is presented here.

### Participants

A total of 166 participants were enrolled in the study; they received course credit for participation and gave informed consent according to the Institutional Review Board policy. Thirty were excluded from analyses because they did not come back for the second session, did not comply with experimental tasks, or because of computer or experimenter error. The remaining 136 participants are reported in the analyses below (blocked training group: *n* = 69, interleaved training group: *n* = 67). Participants were monolingual native speakers of English (i.e., did not have prior exposure to the Hindi sounds) and did not have reading or language disorders.

### Stimuli

Auditory stimuli included five distinct recordings each of the non-words  and  (/u/ vowel context) and  and  (/i/ vowel context). These were produced by a female native speaker of Hindi. All stimuli were presented using Open Sesame (Mathôt, Schreij, & Theeuwes, 2012). Visual stimuli consisted of two Fribbles (novel objects, stimulus images courtesy of Michael J. Tarr, Center for the Neural Basis of Cognition and Department of Psychology, Carnegie Mellon University, http://www.tarrlab.org/) and two novel objects from the Novel Object and Unusual Name (NOUN) database (Horst & Hout, 2016).

### Procedure

Participants came to the lab twice: the first session took place between 5 and 9 PM (evening hours) and the second session took place the following morning (8-10 AM, morning hours). The original study had a 2x2 design that manipulated the training type (blocked vs. interleaved presentation of phonological variability in *training*) and whether participants heard the Hindi sounds (presented word initially) followed by only one of the vowel contexts (/u/ only) or both (/u/ and /i/) in the *assessments* (this manipulation was for assessments only, not the training task, and the motivation for that manipulation is described in detail in the original study). For the purposes of the reanalysis, we are only interested in the manipulation of blocked vs. interleaved training, so we collapse across the vowel context manipulation and only analyze trials with the /u/ vowel context.^2^

Participants completed an AX discrimination task as a pretest, a posttest immediately after training (immediate posttest), and a posttest the following morning (next-day posttest). On each trial, participants heard two of the non-words in a row and were asked to indicate whether they were the same or different.

Participants were trained to learn the Hindi voiced dental and retroflex stop consonants with an identification task. Participants were told they were going to learn new words that corresponded to the novel visual stimuli. Prior to training, participants were familiarized with the correct word–picture pairings. On each trial, participants heard an auditory stimulus and were asked to select the picture that corresponded with the non-word. Both training groups (blocked and interleaved) completed 600 training trials (300 trials for each vowel context). The blocked training group was trained on the /u/ vowel context first, then the /i/ context, and vowel contexts were interleaved for the interleaved group. Participants received visual feedback on every training trial (“Correct”/“Incorrect”). Participants were also tested on identification, but those data are not reported here.

### Analysis approach

Data from the discrimination task are reanalyzed here. First, d’ scores were calculated to account for response bias (Macmillan & Creelman, 2004). To obtain a measure of between-participant variability, the absolute value of the deviation from the group mean was computed for each participant (absolute value (participant d’ – group d’)) at each time point (pretest, immediate posttest, and next-day posttest) and each training group (blocked, interleaved) separately^3^. This measure was inspired by Levene’s test for heterogeneity of variance, as the absolute deviations from the group means are the dependent variable in this test. However, in order to fit a mixed effects model for the data (for repeated measures and in order to be able to generalize to participants outside of the current sample), the absolute deviations were calculated separately and then entered into the model. All data analysis was done in (R Core Team 2020). Models were fit with the lme4 package (Bates et al., 2015), and *p* values were computed with the afex package (Singmann, Bolker, & Westfall, 2019), which uses the Satterthwaite method. All raw data and analysis code are publicly available at https://osf.io/x4erd/.

## Results

To test whether the training groups differed in between-participant variability, a mixed effects model was fit that predicted the absolute values of the deviation from the mean (the dependent variable). Time was included as a fixed effect, which was backwards difference coded using the contr.sdif() function from the MASS package (Venables, 2002) to test the following contrasts: immediate posttest – pretest and next-day posttest – immediate posttest. This will tell us if overall between-participant variability changes over time as a result of training or offline consolidation. Group was included as a fixed factor and was deviation coded (blocked = -.5, interleaved = .5) and was nested within time. Nesting fixed factors allows for tests of simple effects of one factor at each level of the one it is nested within (see Schad, Vasishth, Hohenstein, & Kliegl, 2020). Here the goal is to test for group differences (blocked vs. interleaved) in between-participant variability at each time point.

**Fig. 1:**
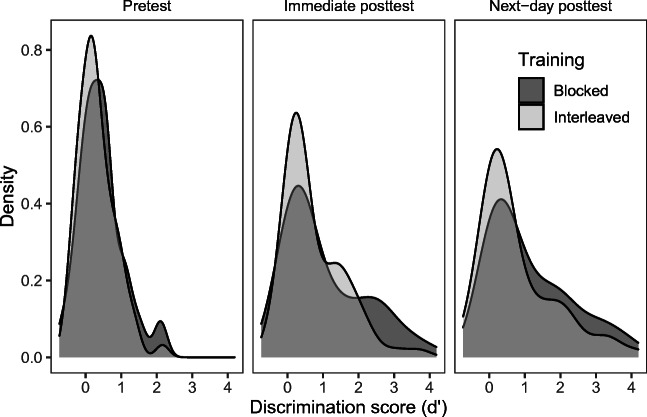
Density plot showing the distribution of raw discrimination scores at each time point for blocked and interleaved training groups to illustrate variability among participants in the two groups. Probability density is shown on the *y*-axis. Density plots can be thought of as a smoothed histogram

Overall, performance on the pretest and immediate posttest differed in between-participant variability, *β* = 0.38 (95% CI [0.29, 0.48]), *SE* = .05, *t* = 7.97, *p* <.001, but the difference in variability between the next-day posttest and immediate posttests was smaller, *β* = 0.09 (95% CI [0.00, 0.19]), *SE* = .05, *t* = 1.87, *p* = .06. The blocked and interleaved training groups showed almost no difference in variability at pretest, which is expected, *β* = -0.07 (95% CI [-0.25, 0.11]), *SE* = .09, *t* = -.72, *p* = .47. However, they did differ in variability at the immediate posttest, *β* = -0.28 (95% CI [-0.45, -0.10]), *SE* = .09, *t* = -2.99, *p* = .003. Unexpectedly, the sign of the coefficient indicates that the blocked training group had higher variability than the interleaved group. The group differences in variability are much smaller at the next-day posttest after a period of offline consolidation, *β* = -0.14 (95% CI [-0.32, 0.04]), *SE* = .09, *t* = -1.52 *p* = .13, see Fig. 1).

## Discussion

The current study presented a reanalysis of the discrimination task from Fuhrmeister and Myers (2020) to test for group differences in between-participant variability as a result of blocked or interleaved phonetic training. In the original study, participants were trained to learn the Hindi voiced dental and retroflex stop consonants and heard the sounds in two vowel contexts during training (/i/ and /u/), presented in either a blocked or interleaved manner. The two groups were tested at three time points: before training (pretest), immediately after training (immediate posttest), and again the next morning after an approximate 12-h interval containing sleep (next-day posttest). The original study found no significant differences in means between the two training groups on a discrimination task. Previous research suggests that high-aptitude individuals typically benefit from interleaved phonetic training, whereas more learners can benefit from blocked training, even those with poorer aptitude for the task (Perrachione et al., 2011). Because of these findings, we predicted that interleaved training would result in more between-participant variability because high-aptitude learners would show a disproportional benefit from this (likely more difficult) type of training. Instead, we found the opposite pattern: the blocked training group showed more between-participant variability, at least immediately after training. The density plot in Fig. 1 shows that the blocked training group had a larger proportion of learners with higher scores than the interleaved group at the immediate posttest, but the two groups show a more similar distribution of scores after a delay.

There are several differences in the present study and previous studies, such as Perrachione, Lee, Ha, and Wong (2011), that found that high-aptitude learners benefitted from interleaved training, and these differences may explain why the predictions for the current study were not borne out. First, studies by Perrachione et al., (2011) and Sadakata and McQueen (2014) trained participants to learn non-native tones, which did not exist in the participants’ first language phonological inventory. The Hindi speech sound contrast used in the present study is known to be especially challenging for native speakers of English because learners already have a very similar category (alveolar /d/) in their first-language phonetic inventory. This perceptually similar native-language category encompasses dental and retroflex variants as allophones (e.g., in “width” or “address”), and learning to distinguish two sounds that map to one first-language speech category is particularly difficult (e.g., Best, McRoberts, & Goodell, 2001; Best, McRoberts, & Sithole, 1988). It is possible that the stimuli in the current study were quite difficult, and only some participants were able to benefit from training immediately and only from the easier training design. As discussed in the Introduction, the present study included much less stimulus variability compared to previous studies, as well as fewer training sessions, and all of these variables could have contributed to why the results were not what was predicted.

The current results indicate that the differences in participant variability between the groups decreased after a period of offline consolidation, which raises the question whether the group differences at the immediate posttest are important if they go away that quickly. Reducing between-participant variability and identifying equalizing treatments may be desirable for learning second-language speech sounds outside the laboratory, for example, in classroom settings. In the current study, learners were exposed to a very minimal amount of variability in the speech signal in training (one talker produced the consonants to be learned in two phonological contexts). For practical purposes, it may not matter whether stimuli are blocked or interleaved when learners hear only limited amounts of variability in training. However, the acoustic speech signal contains much more variability than was presented in this study, and it is important to better understand how variability can be presented in training to achieve the best possible outcomes for second language speech perception and production. It may be of interest in the future to test for group differences in variance with other training designs that include more talker and phonological variability (e.g., high-variability training) and that test for differences over a longer period of time.

Nonetheless, there are theoretical and methodological reasons to test for differences in either means or variance at different time points throughout the learning trajectory. First, some theories make specific predictions about the time course of learning, and testing for differences in means or variance at different time points allows us to test predictions from these theories to see whether they hold for non-native speech sound learning. Specifically, the decrease in between-participant variability after sleep/offline consolidation has both theoretical and practical relevance for the role of sleep for non-native speech sound learning. Previous studies have shown that participants improve their perception of non-native contrasts after sleep (Earle & Myers, 2015; Qin & Zhang, 2019), and the current study suggests that sleep or a period of offline consolidation may additionally act as an equalizing factor among participants for non-native speech sound training paradigms. This issue also has methodological relevance. Some studies of non-native speech sound learning (and perceptual learning of speech more generally) test for learning within a single session. For such studies, it is important to acknowledge the possibility that some group differences or effects of interest may not show up until later or may disappear even after a short delay, for example, after memory traces have been consolidated offline or during sleep.

The reanalysis presented here suggests that examining between-participant variability as an outcome variable may prove useful in a field where between-participant variability is so common, and this may give us more information than we can glean from examining means alone. This approach could also have methodological relevance for identifying variables that are associated with successful non-native speech sound learning. Several studies to date have utilized a correlational approach to test whether certain skills are related to non-native speech sound learning and these studies have identified many skills that contribute to better speech sound learning in both perception and production. Some of these include individual differences in phonological skills (Fuhrmeister, Schlemmer, & Myers, 2020; Earle & Arthur, 2017), acoustic cue weighting (Schertz, Cho, Lotto, & Warner, 2015), and even attitudes towards a speaker and personality traits, such as openness to new experiences (Yu, Abrego-Collier, & Sonderegger, 2013). However, training paradigms may differ in how much heterogeneity of variance they produce. Paradigms that result in less heterogeneity of variance (i.e., “equalizing treatments”) may be preferable for “real-word” learning, but it is easier to find correlations between two variables if participants perform quite differently on the tasks (i.e., these tasks can more easily rank participants in their performance on them, Hedge, Powell, & Sumner, 2018). Analyzing group differences in between-participant variability could complement correlational approaches to identify training paradigms that will be most promising for identifying relationships between non-native speech sound learning and other variables.

### Conclusions

Studies on non-native speech sound learning are notorious for reporting large amounts of between-participant variability, and this variability provides opportunities to learn more about the underlying processes involved in speech sound learning. The present reanalysis provides an example of how explicit testing of group differences in between-participant variability in addition to means may alter our interpretation of findings or give us a more complete picture of the effects being tested. Examining between-participant variability may be a crucial step in uncovering whether patterns of individual variability in non-native speech sound learning are systematic. This could be a useful tool in evaluating the efficacy of specific training paradigms or for research on the potential sources of individual variability in non-native speech sound learning.

## Footnotes

1 Data from a two-alternative forced choice identification task were also collected, but participants typically show less inter-individual variability on that task (likely due to ceiling effects), so those data are not presented here.

2 See the OSF repository at https://osf.io/x4erd/ for a reanalysis of between-participant variability with the original 2x2 design; the pattern of results does not change from what is reported here.

3 Note that the pattern of results does not change when deviations from the group means are calculated for each of the subgroups from the original 2x2 design.
